# Early non-contrast CT morphology at emergency admission in acute pancreatitis: real-world associations with clinical course

**DOI:** 10.1007/s10140-026-02475-1

**Published:** 2026-05-11

**Authors:** Bartosz Molasy, Wiktoria Zawolik-Kosek, Agata Gronowska, Stanisław Głuszek

**Affiliations:** 1 Medical College, The Jan Kochanowski University, Kielce, Poland; 2 Department of General Surgery, St Alexander Hospital, Kielce, Poland; 3 Department of General Surgery, Ministry of Internal Affairs and Administration in Kielce, Independent Public Health Care Institution, Kielce, Poland; 4 Department of Surgical Oncology, Holy Cross Cancer Center, Kielce, Poland; 5 Institute of Genetics and Animal Biotechnology, Polish Academy of Sciences, Magdalenka, Poland

**Keywords:** Acute pancreatitis, Imaging pathways, Ultrasound-first, Non-contrast CT, Balthazar classification

## Abstract

**Purpose:**

To evaluate the clinical associations of admission non-contrast CT morphology in acute pancreatitis within a real-world emergency workflow.

**Materials and methods:**

This retrospective observational cohort study included 264 consecutive adult patients admitted with acute pancreatitis to two surgical centers between 2019 and 2024. Patients were categorized according to the first imaging modality obtained at admission into an ultrasound-first (US-first) or computed tomography–first (CT-first) pathway. Baseline characteristics and in-hospital outcomes were compared between pathways. In the CT-first subgroup, all examinations were performed without intravenous contrast, and morphologic severity was assessed using the Balthazar classification. Associations between CT morphology and clinical outcomes were evaluated using univariable analyses.

**Results:**

Of the 264 patients, 143 (54.2%) were managed within a US-first pathway and 121 (45.8%) underwent CT as the initial imaging modality. Baseline demographic and etiologic characteristics were comparable between pathways. Patients in the CT-first pathway demonstrated numerically higher rates of adverse clinical outcomes at admission, including a longer length of hospital stay (median 8 vs. 6 days; *p* = 0.01) and numerically higher rates of severe acute pancreatitis and in-hospital mortality.

Within the CT-first cohort, non-contrast CT morphology demonstrated heterogeneous inflammatory severity. Higher Balthazar grades were associated with stepwise numerical increases in rates of severe disease, complications, and length of hospital stay. When dichotomized, advanced morphologic severity (Balthazar grades D–E) showed higher odds of adverse outcomes compared with grades A–C, although these associations did not reach statistical significance.

**Conclusion:**

In routine emergency practice, selection of ultrasound-first or CT-first imaging pathways appears largely driven by triage and organizational factors rather than predefined imaging strategies. In patients undergoing non-contrast CT at admission, higher Balthazar grades demonstrated consistent numerical gradients toward more severe clinical courses; however, these associations did not reach statistical significance. Early non-contrast CT morphology should therefore be interpreted as contextual inflammatory assessment rather than a standalone prognostic tool.

## Introduction

Acute pancreatitis (AP) is one of the most common causes of emergency hospital admission for acute abdominal pain worldwide and represents a substantial clinical and organizational burden for emergency departments and surgical units [1, 2]. Despite advances in supportive care, intensive monitoring, and minimally invasive interventions, mortality in severe forms of AP remains high, reported between 15% and 30% in contemporary cohorts [3–5]. Early diagnostic assessment is therefore critical, as decisions made during the first hours of admission directly influence patient triage, monitoring intensity, escalation of imaging, and allocation of hospital resources [4–6].

The Revised Atlanta Classification provides the currently accepted clinical and morphological framework for acute pancreatitis, stratifying disease severity into mild, moderately severe, and severe categories based on the presence and duration of organ failure and local or systemic complications [1, 7]. Imaging plays a central role within this framework, supporting diagnostic confirmation, etiologic assessment, and evaluation of disease extent and complications [7, 8]. International guidelines issued by the American College of Gastroenterology (ACG), and the American Gastroenterological Association (AGA), consistently recommend abdominal ultrasonography (US) as the first-line imaging modality in patients with suspected acute pancreatitis [2, 9]. Ultrasonography enables rapid bedside confirmation of the diagnosis, identification of biliary etiology through detection of gallstones or bile duct dilatation, and exclusion of alternative causes of acute abdomen, while avoiding ionizing radiation and contrast exposure [6, 10, 11].

Accordingly, current guidelines discourage routine early CT and recommend its use selectively in cases of diagnostic uncertainty, suspected complications, or clinical deterioration [2, 4, 6]. In real-world practice, however, adherence to an ultrasound-first strategy is frequently influenced by organizational and logistical constraints rather than guideline intent alone [8, 12, 13]. Continuous 24-hour availability of experienced sonographers or radiologists is not guaranteed in many hospitals, particularly during night shifts and weekends, even in centers providing emergency surgical care [10, 12]. When ultrasonography cannot be obtained promptly, CT is often performed as the initial imaging modality to rapidly confirm the diagnosis and exclude other urgent intra-abdominal pathology [8, 10, 11].

In many emergency settings, CT examinations in suspected acute pancreatitis are routinely performed without intravenous contrast because of renal impairment, hemodynamic instability, contrast contraindications, or time constraints [8, 14, 15]. In this context, radiologic assessment relies primarily on morphologic inflammatory changes rather than enhancement-based evaluation of pancreatic necrosis [14, 15]. The Balthazar morphologic classification provides a standardized and reproducible framework for grading these changes without requiring contrast administration [16].

The Balthazar classification represents the morphologic component of the CT Severity Index but can also be applied independently when necrosis assessment is not feasible [16–18]. Higher Balthazar grades have been associated with increased risk of organ failure, local complications, prolonged hospitalization, and mortality, even in cohorts where contrast-enhanced imaging was not systematically available [14, 17–19]. Although isolated morphologic grading is less comprehensive than composite indices incorporating necrosis extent, it remains clinically meaningful and closely aligned with real-world radiologic reporting at admission [14, 15, 19].

From a radiologic perspective, ultrasound-first and CT-first pathways therefore address fundamentally different diagnostic questions [6, 13]. Ultrasound-first imaging primarily supports diagnosis and etiologic assessment, whereas CT-first imaging—particularly when performed without contrast—functions as a triage tool to assess inflammatory burden and exclude alternative diagnoses under emergency conditions [6, 8]. Understanding their relationship to clinical outcomes thus requires a focus on workflow and organizational determinants rather than modality performance [12, 13].

The primary aim of this study was to evaluate whether inflammatory morphology on admission non-contrast CT is associated with subsequent clinical course in a real-world emergency cohort of patients with acute pancreatitis. A secondary aim was to describe how initial imaging pathways reflect organizational workflow rather than predefined imaging strategies.

## Materials and methods

### Study design and setting

This retrospective observational cohort study was conducted in two surgical centers providing emergency care for patients with acute abdominal conditions. Consecutive adult patients admitted with a diagnosis of acute pancreatitis between January 2019, and December 2024 were screened for eligibility. The study was approved by the local institutional bioethics committee (approval No. 1/2024) and conducted in accordance with the Declaration of Helsinki.

## Study population

Acute pancreatitis was diagnosed according to the Revised Atlanta Classification and required the presence of at least two of the following three criteria:

1. Characteristic abdominal pain consistent with acute pancreatitis.
2. Serum amylase and/or lipase activity at least three times the upper limit of normal.
3. Imaging findings consistent with acute pancreatitis on ultrasonography or computed tomography.

Exclusion criteria included chronic pancreatitis, pancreatic malignancy, post-traumatic or postoperative pancreatitis, and incomplete clinical or imaging documentation precluding classification of the initial imaging pathway or assessment of clinical outcomes. After application of these criteria, a total of 264 patients constituted the final study cohort.

## Imaging pathways and cohort flow

Patients were categorized according to the initial imaging modality performed during the diagnostic work-up at admission. Two imaging pathways were defined:

- US-first pathway: patients in whom abdominal ultrasonography was the first imaging examination obtained after presentation.
- CT-first pathway: patients in whom computed tomography was the first imaging examination obtained at admission.

The choice of the initial imaging modality was neither protocolized nor randomized. In both participating centers, abdominal ultrasonography is recommended as the first-line imaging modality for suspected acute pancreatitis. However, continuous 24-hour availability of an experienced radiologist or sonographer is not guaranteed, particularly during night shifts and weekends. When ultrasonography could not be performed promptly due to organizational constraints, computed tomography was frequently used as the initial imaging modality to confirm the diagnosis and exclude alternative causes of acute abdominal pain.

Accordingly, assignment to the US-first or CT-first pathway primarily reflects organizational availability and real-world triage logistics rather than a predefined clinical decision or imaging strategy. Subsequent imaging examinations performed during hospitalization did not affect pathway classification, which was based exclusively on the first imaging modality obtained at admission.

### Ultrasonography

Abdominal ultrasonography was performed using standard high-resolution ultrasound systems equipped with convex transducers (frequency range 3.5–5.0 MHz). Examinations were conducted by experienced radiologists or supervised trainees in accordance with routine clinical practice.

Pancreatic appearance on ultrasound was categorized as normal, edematous, non-visualized, or other, and the presence of peripancreatic fluid collections was recorded as a binary variable. Ultrasound findings were recorded descriptively and used to support diagnostic confirmation and identification of biliary etiology. In keeping with the study focus on imaging pathways rather than diagnostic performance, ultrasound findings were not incorporated into predictive modeling or multivariable analyses. Ultrasound assessment was recorded descriptively as part of routine clinical documentation and was not standardized for research purposes.

### Computed tomography (non-contrast)

Computed tomography was performed without intravenous contrast in all patients assigned to the CT-first pathway. Non-contrast CT was used as part of the initial diagnostic evaluation to assess pancreatic morphology and exclude alternative intra-abdominal pathology. Because intravenous contrast was not administered, pancreatic necrosis could not be assessed and necrosis-based indices such as CTSI were not calculated. CT examinations were were reviewed as part of routine clinical reporting by board-certified radiologists and were performed using multidetector scanners with standard abdominal acquisition protocols and slice thickness of 3–5 mm.

CT examinations were reviewed for pancreatic enlargement, contour irregularity, peripancreatic inflammatory changes, and the presence of peripancreatic fluid collections. Based on these morphologic features, CT severity was characterized using the Balthazar morphologic classification, ranging from grade A (normal pancreas) to grade E (extensive peripancreatic inflammatory changes or collections).

For selected analyses, Balthazar grades D and E were grouped as a binary high-risk category for analytic purposes, reflecting advanced local inflammatory involvement. Because contrast enhancement was not used, pancreatic necrosis could not be reliably assessed, and composite CT-based severity indices requiring necrosis quantification, such as the CT Severity Index (CTSI) or modified CTSI, were not calculated. Accordingly, the present analysis focused exclusively on morphologic inflammatory changes visible on non-contrast CT.

### Clinical data and outcomes

Clinical data were extracted from electronic medical records and included age, sex, comorbidities, etiology of acute pancreatitis, length of hospital stay, and in-hospital outcomes. Biliary etiology was defined based on the final clinical diagnosis, including gallstones, biliary sludge, or transient biliary obstruction, and was not limited to gallstones visualized on admission ultrasound.

The primary outcomes were:

1. Severe acute pancreatitis, defined according to the Revised Atlanta Classification.
2. Occurrence of complications, including local pancreatic and peripancreatic complications (acute peripancreatic fluid collections, acute necrotic collections, pseudocysts, and walled-off necrosis) as well as documented systemic complications during hospitalization.
3. In-hospital mortality.

Length of hospital stay (LOS), defined as the number of days from admission to discharge or death, was analyzed as a secondary outcome.

### Statistical analysis

Continuous variables are presented as median with interquartile range (IQR), and categorical variables as counts and percentages. Differences between the US-first and CT-first groups were assessed using the Mann–Whitney U test for continuous variables and the χ² test or Fisher’s exact test for categorical variables, as appropriate.

Analyses evaluating the association between CT morphology and clinical outcomes were restricted to the CT-first subgroup. Associations between Balthazar grade and outcomes were assessed using univariable regression analyses. Effect estimates are reported as odds ratios with 95% confidence intervals. No multivariable adjustment was performed due to the limited number of patients with high Balthazar grades (D–E) and outcome events, to avoid model overfitting.

All statistical tests were two-sided, and a p-value < 0.05 was considered statistically significant. Statistical analyses were performed using R software (R Foundation for Statistical Computing, Vienna, Austria).

## Results

### Study population and imaging pathways

**Fig. 1:**
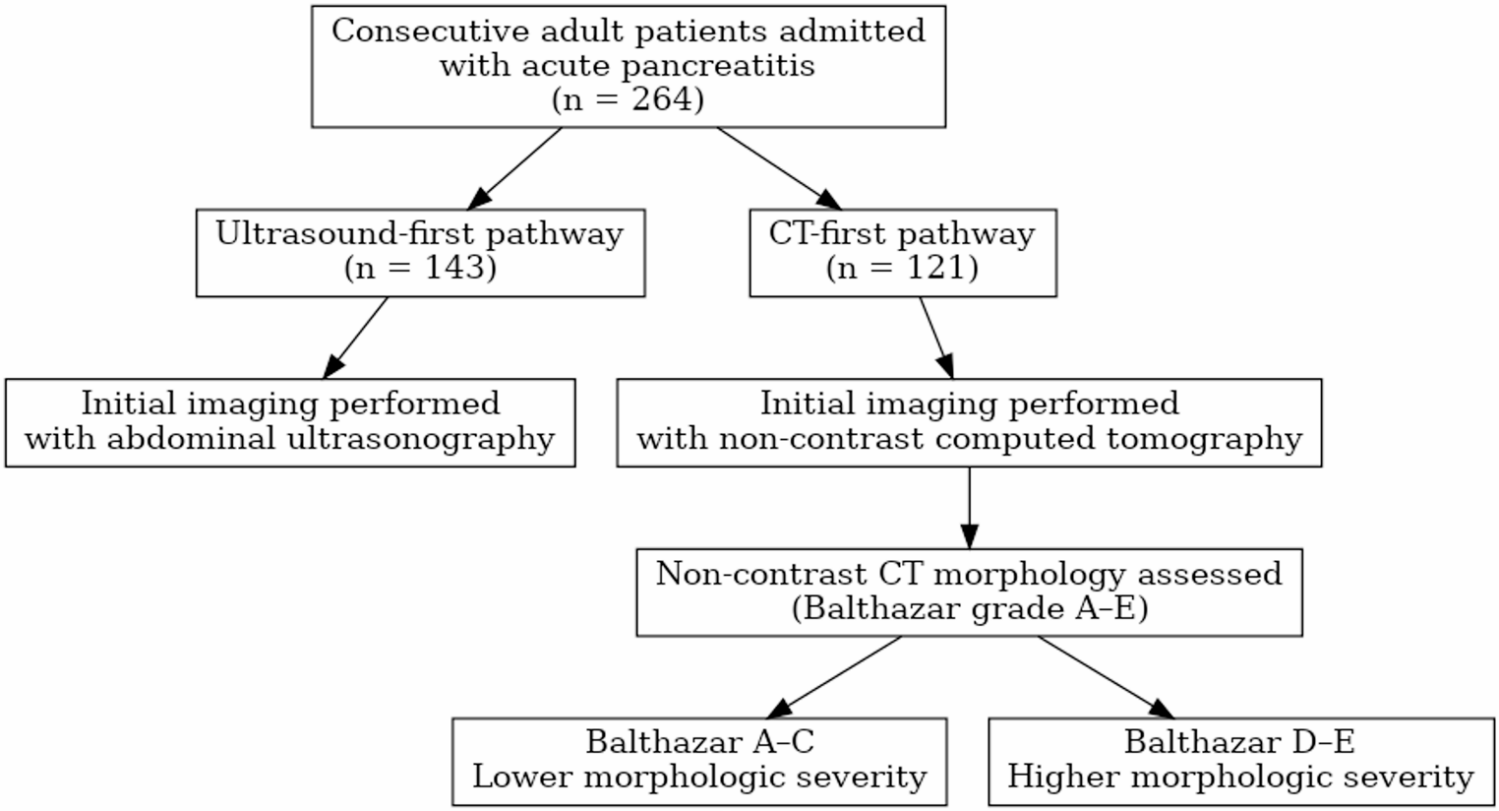
Cohort flow and real-world imaging pathways in acute pancreatitis. A total of 264 consecutive patients admitted with acute pancreatitis were included in the study. Patients were categorized according to the initial imaging modality performed at admission into an ultrasound-first (US-first) pathway or a computed tomography–first (CT-first) pathway. Allocation to imaging pathways was not randomized and primarily reflected organizational availability of ultrasonography. In patients undergoing CT-first imaging, all examinations were performed without intravenous contrast, and morphologic severity was assessed using the Balthazar classification (grades A–E)

**Fig. 2:**
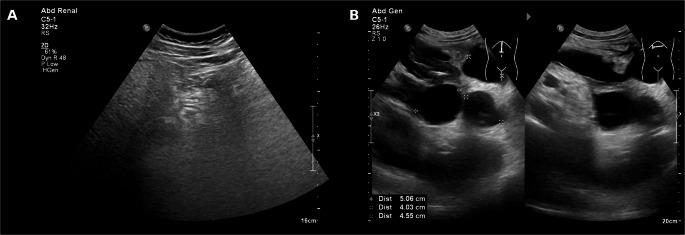
Representative ultrasound findings in acute pancreatitis. (**A**) Abdominal ultrasound with limited visualization of the pancreas due to overlying bowel gas, illustrating a common limitation of ultrasonography in emergency imaging. (**B**) Abdominal ultrasound demonstrating peripancreatic fluid collections (anechoic areas) adjacent to the pancreas in a patient with acute pancreatitis

A total of 264 consecutive adult patients admitted with a diagnosis of acute pancreatitis were included in the final analysis. According to the initial imaging modality performed during the diagnostic work-up at admission, 143 patients (54.2%) were managed within an ultrasound-first (US-first) imaging pathway, whereas 121 patients (45.8%) underwent computed tomography as the first imaging examination (CT-first pathway), as illustrated in Fig. 1. In the US-first pathway, abdominal ultrasound was the initial imaging modality at admission. In a substantial proportion of examinations, visualization of the pancreas was limited due to overlying bowel gas or patient body habitus, reflecting common real-world limitations of emergency ultrasonography. Representative ultrasound findings from the study cohort are shown in Fig. 2.

Baseline demographic and clinical characteristics stratified by imaging pathway are summarized in Table 1. The median age of the overall cohort was 56 years (IQR 40–75), with a balanced sex distribution (51.5% male). The prevalence of major comorbidities, including obesity, type 2 diabetes mellitus, and active smoking, did not differ significantly between the two pathways. Similarly, the etiologic distribution of acute pancreatitis was comparable between groups, with biliary and alcohol-related disease accounting for most cases in both pathways. Despite broadly similar baseline characteristics, patients managed within the CT-first pathway demonstrated numerically higher rates of adverse outcomes at admission. Median length of hospital stay was significantly longer in the CT-first group compared with the US-first group (8 vs. 6 days; *p* = 0.01). Patients undergoing CT-first imaging demonstrated numerically higher rates of adverse outcomes, consistent with differences in early clinical context rather than an effect of the imaging modality itself.

**Table 1:** Baseline characteristics of patients according to initial imaging pathway

Characteristic	Overall cohort (*n* = 264)	US-first pathway (*n* = 143)	CT-first pathway (*n* = 121)	*p* value
Age, years	56 (40–75)	55 (39–72)	58 (42–77)	0.29
Male sex, n (%)	136 (51.5)	70 (49.0)	66 (54.5)	0.46
Body mass index ≥ 30 kg/m², n (%)	97 (36.7)	51 (35.7)	46 (38.0)	0.77
Type 2 diabetes mellitus, n (%)	38 (14.4)	18 (12.6)	20 (16.5)	0.42
Current smoker, n (%)	97 (36.7)	51 (35.7)	46 (38.0)	0.81
Etiology of acute pancreatitis, n (%)				
– Biliary	85 (32.2)	49 (34.3)	36 (29.8)	
– Alcohol-related	94 (35.6)	43 (30.1)	51 (42.1)	
– Idiopathic	63 (23.9)	36 (25.2)	27 (22.3)	
– Other	22 (8.3)	15 (10.5)	7 (5.8)	
C-reactive protein, mg/L	13.7 (3.7–73.5)	12.1 (3.2–65.8)	17.8 (5.4–84.3)	0.19
Length of hospital stay, days	7 (5–10)	6 (4–9)	8 (5–12)	0.01
Severe acute pancreatitis, n (%)	32 (12.1)	13 (9.1)	19 (15.7)	0.058
Any complications, n (%)	41 (15.5)	20 (14.0)	21 (17.4)	0.50
In-hospital mortality, n (%)	18 (6.8)	8 (5.6)	10 (8.3)	0.47

Data are presented as median (interquartile range) or number (percentage). Percentages are calculated within each column. Comparisons between groups were performed using the Mann–Whitney U test for continuous variables and the χ² test or Fisher’s exact test for categorical variables, as appropriate

Severe acute pancreatitis, as defined by the Revised Atlanta Classification, occurred in 32 patients (12.1%) in the overall cohort. When stratified by imaging pathway, severe disease was observed more frequently among patients managed within the CT-first pathway compared with those in the US-first pathway (15.7% vs. 9.1%, respectively). Although this difference did not reach statistical significance (*p* = 0.058), the numerical imbalance indicates a tendency toward more severe clinical presentation among patients undergoing CT as the initial imaging modality.

Local or systemic complications developed in 41 patients (15.5%) during hospitalization. The overall incidence of complications was comparable between imaging pathways, occurring in 14.0% of patients managed with a US-first strategy and 17.4% of patients managed with a CT-first strategy (*p* = 0.50). In-hospital mortality for the entire cohort was 6.8%, corresponding to 18 deaths, and was numerically higher in the CT-first pathway compared with the US-first pathway (8.3% vs. 5.6%), although this difference was not statistically significant (*p* = 0.47).

### Non-contrast CT morphology and distribution of Balthazar grades

All patients managed within the CT-first pathway (*n* = 121) underwent non-contrast computed tomography at admission. Image quality was sufficient for morphologic assessment in all cases, allowing reliable classification according to the Balthazar grading system.

**Fig. 3:**
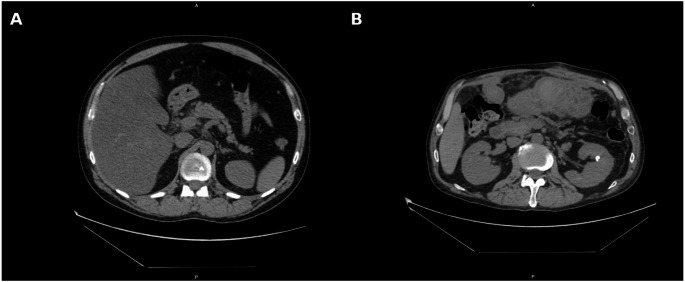
Non-contrast CT morphology in acute pancreatitis. (**A**) Axial non-contrast CT image showing pancreatic enlargement and peripancreatic inflammatory changes corresponding to Balthazar grade C. (**B**) Axial non-contrast CT image demonstrating extensive peripancreatic inflammatory changes and fluid collections consistent with Balthazar grade E

The distribution of Balthazar grades demonstrated substantial heterogeneity in morphologic severity across the CT-first cohort. Grade A morphology was observed in 21 patients (17.4%), grade B in 35 patients (28.9%), and grade C in 49 patients (40.5%). More advanced inflammatory changes were present in 6 patients (5.0%) classified as grade D and in 10 patients (8.3%) classified as grade E. Overall, 16 patients (13.2%) demonstrated high morphologic severity (Balthazar grades D–E), whereas the majority of the cohort (105 patients, 86.8%) exhibited low-to-moderate morphologic severity (grades A–C). Representative examples of CT morphology observed in the cohort, including moderate (Balthazar C) and severe inflammatory changes (Balthazar E), are presented in Fig. 3.

### Relationship between CT morphology and disease severity

A progressive increase in disease severity was observed across increasing Balthazar grades. Severe acute pancreatitis occurred in 14.3% of patients with grade A morphology and in 8.6% of patients with grade B morphology. The proportion increased to 16.3% among patients with grade C changes and remained elevated in grade D (16.7%), reaching the highest rate in patients with grade E morphology (40.0%).

When dichotomized (Balthazar grades D–E vs. A–C), patients with higher morphologic severity demonstrated numerically higher odds of severe acute pancreatitis compared with those with lower-grade morphology (OR 2.73; 95% CI 0.83–8.97); however, this association did not reach statistical significance (*p* = 0.14).

### CT morphology and complications

The incidence of complications increased with advancing morphologic severity. Complications occurred in 9.5% of patients with grade A morphology and in 5.7% of patients with grade B morphology, rising to 26.5% among patients with grade C changes. In patients with advanced inflammatory changes, complication rates increased further, reaching 33.3% in grade D and 20.0% in grade E.

When analyzed as a binary variable (Balthazar grades D–E vs. A–C), higher morphologic severity was associated with increased odds of complications (OR 1.73; 95% CI 0.50–5.99); however, this association did not reach statistical significance (*p* = 0.48).

### CT morphology, mortality, and length of hospital stay

In-hospital mortality demonstrated a stepwise numerical increase with advancing morphologic severity, although absolute event numbers were small. Mortality occurred in 9.5% of patients with grade A morphology and in 2.9% of patients with grade B morphology, increased to 10.2% in grade C, and reached 16.7% in grade D, while patients with grade E morphology exhibited a mortality rate of 10.0%. When analyzed as a binary outcome, the association between high morphologic severity and mortality did not reach statistical significance.

**Fig. 4:**
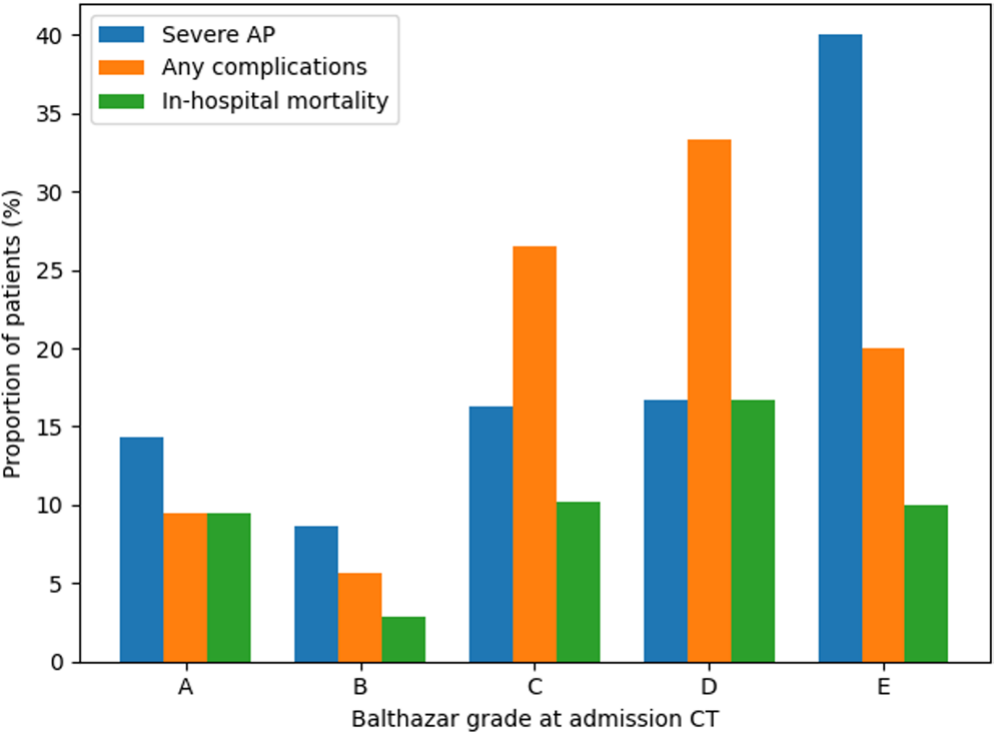
Clinical outcomes according to Balthazar morphologic grade on non-contrast CT at admission

Length of hospital stay increased progressively with higher Balthazar grades, indicating an association between admission CT morphology and subsequent hospital resource utilization. A comprehensive summary of clinical outcomes according to Balthazar morphologic grade is presented in Table 2; Fig. 4.

**Table 2:** Clinical outcomes according to Balthazar morphologic grade in the CT-first pathway (*n* = 121)

Balthazar grade	Patients, *n*	Severe acute pancreatitis, *n* (%)	Any complications, *n* (%)	In-hospital mortality, *n* (%)
A	21	3 (14.3)	2 (9.5)	2 (9.5)
B	35	3 (8.6)	2 (5.7)	1 (2.9)
C	49	8 (16.3)	13 (26.5)	5 (10.2)
D	6	1 (16.7)	2 (33.3)	1 (16.7)
E	10	4 (40.0)	2 (20.0)	1 (10.0)
Total	121	19 (15.7)	21 (17.4)	10 (8.3)

Data are presented as number (percentage). Percentages are calculated within each Balthazar grade. Severe acute pancreatitis was defined according to the Revised Atlanta Classification. CT examinations were performed without intravenous contrast in all patients

## Discussion

In this real-world observational study, initial imaging pathways in acute pancreatitis reflected early clinical triage rather than random allocation, with patients undergoing CT-first imaging demonstrating a more severe overall clinical profile at admission. Within the CT-first pathway, non-contrast CT morphology assessed using the Balthazar classification showed consistent numerical trends toward higher rates of severe disease, complications, and longer hospital stay with increasing morphologic severity.

Increasing morphologic severity correlated with higher rates of severe acute pancreatitis, development of complications, and prolonged length of stay. These findings suggest that, even in the absence of contrast enhancement, early CT morphology reflects inflammatory burden and shows consistent gradients across clinical outcomes.

### Imaging pathways as markers of real-world triage

Current international guidelines consistently recommend abdominal ultrasonography as the first-line imaging modality in suspected acute pancreatitis, primarily to confirm the diagnosis, identify biliary etiology, and exclude alternative causes of acute abdomen [1, 2, 4, 6]. Early routine CT is discouraged, as it rarely alters management in mild disease and may underestimate necrosis when performed within the first 48–72 h [2–4]. Nevertheless, real-world adherence to an ultrasound-first strategy is strongly influenced by organizational factors, including the availability of experienced sonographers, time of admission, and emergency department workflow [8, 10, 11, 13].

Our data illustrate how these organizational realities translate into distinct imaging pathways. CT-first imaging was frequently employed during periods of limited ultrasound availability, capturing patients who were clinically perceived as more severe or diagnostically uncertain. Similar observations have been reported in prior real-world and emergency medicine–oriented studies, where CT-first imaging often reflects triage urgency rather than deliberate deviation from guidelines [8, 12, 13]. From a radiologic perspective, this distinction is critical: outcome differences between imaging pathways should not be interpreted as evidence for or against a given modality, but rather as reflections of patient selection and workflow.

By explicitly framing ultrasound-first and CT-first approaches as organizationally driven pathways, our study avoids a common interpretative pitfall in retrospective imaging research. Rather than comparing diagnostic performance, we emphasize how imaging choice functions as a surrogate marker of early clinical context, a perspective increasingly advocated in contemporary radiology literature [11, 13, 14]. Therefore, differences in outcomes between imaging pathways should be interpreted as reflecting confounding by indication and triage-related selection rather than any intrinsic superiority of one imaging modality over the other.

### Value of non-contrast CT morphology at admission

The present findings apply exclusively to non-enhanced CT performed at admission. Morphologic grading reflects inflammatory extent but does not evaluate perfusion or necrosis. Therefore, these results should not be interpreted as a substitute for contrast-enhanced CT in definitive severity assessment. In many emergency settings, intravenous contrast is deferred due to renal dysfunction, hemodynamic instability, or time constraints [10, 15]. Consequently, radiologists are frequently required to provide clinically actionable assessments based solely on morphologic inflammatory changes.

Importantly, although higher Balthazar grades were associated with numerically increased rates of severe acute pancreatitis and complications, these associations did not consistently reach statistical significance when analyzed in a dichotomized manner. This likely reflects the relatively small number of patients with advanced morphologic severity (grades D–E) in the present cohort, a limitation inherent to real-world emergency imaging populations.

Nevertheless, the observed stepwise gradients across individual Balthazar grades suggest that early non-contrast CT morphology reflects inflammatory extent and aligns with subsequent clinical course, although it does not establish independent prognostic value. These results are consistent with earlier studies demonstrating that the morphologic component of CT-based scoring systems remains prognostically informative, even when necrosis cannot be directly assessed [16–19].

While composite indices such as CTSI or modified CTSI outperform isolated morphology in comprehensive prognostic modeling, they rely on contrast enhancement and are therefore not universally applicable at admission [20, 21]. Our data suggest a pragmatic role for morphologic grading as a rapid, reproducible tool that aligns with real-world CT protocols and reporting practices, particularly in the early triage phase.

### Contextualizing mortality and limitations of morphologic prediction

The association between Balthazar grade and mortality in our cohort followed a clear stepwise trend but did not reach statistical significance in binary analyses. This finding aligns with contemporary understanding that mortality in acute pancreatitis is driven by multifactorial mechanisms, including systemic inflammatory response, organ failure, comorbidities, and infection, which are not fully captured by morphologic imaging alone [4, 6, 22].

Recent reviews emphasize that imaging-based scores should be interpreted as complementary rather than definitive prognostic tools [14, 22, 23]. In this context, our results reinforce a nuanced role for non-contrast CT: it reflects inflammatory burden and shows consistent associations with complication rates but should not be interpreted as a standalone predictor of survival.

### Ultrasound, CT, and the evolving multimodality landscape

Ultrasonography remains indispensable for etiologic assessment, particularly in biliary pancreatitis, but its limitations in pancreatic visualization are well documented [9, 24, 25]. CT offers superior anatomic delineation and complication detection, while MRI provides added value in selected cases, especially for characterization of complex fluid collections and follow-up imaging [26–29]. Recent comparative and review studies highlight the complementary rather than competitive roles of these modalities [25, 26, 28, 30].

Our study deliberately avoided direct performance comparisons between ultrasound and CT, as such analyses would be heavily confounded by operator dependence, timing, and pathway selection. Instead, we focused on how each modality functions within a real-world workflow, an approach increasingly encouraged in radiology research addressing emergency imaging pathways [13, 23].

### Clinical and radiologic implications

From a clinical and radiologic perspective, these findings suggest that non-contrast CT morphology at admission may contribute to early contextual assessment during emergency triage where contrast-enhanced imaging may be delayed or contraindicated. Rather than serving as a standalone predictor of adverse outcomes, morphologic severity appears to provide contextual information that complements early clinical assessment and laboratory data.

More broadly, this study highlights the need to interpret imaging findings within the organizational and clinical context in which they are obtained. As imaging pathways continue to evolve alongside advances in AI-assisted analysis and multimodality integration, understanding real-world workflows will remain essential for translating radiologic information into meaningful clinical impact [22, 29–31].

### Limitations and future directions

This study has several limitations that should be considered when interpreting the findings. The retrospective design and the absence of protocolized imaging pathways introduce the possibility of confounding by indication. Allocation to ultrasound-first or CT-first pathways largely reflected real-world triage decisions, diagnostic uncertainty, and local organizational factors rather than randomized assignment. Consequently, pathway-related differences should not be interpreted causally or as evidence of superiority of one imaging modality over another.

All CT examinations were performed without intravenous contrast, which precluded assessment of pancreatic necrosis and calculation of necrosis-based severity indices such as the CT Severity Index (CTSI) or modified CTSI. The present findings therefore apply specifically to inflammatory morphologic grading on non-contrast CT at admission and cannot replace contrast-enhanced CT for definitive evaluation of pancreatic necrosis.

The number of patients with advanced morphologic severity (Balthazar grades D–E) was relatively small, resulting in wide confidence intervals and limited statistical power in dichotomized analyses. Although consistent numerical gradients were observed across outcomes, several associations did not reach statistical significance and should therefore be interpreted as exploratory.

CT examinations were interpreted as part of routine clinical reporting, and formal inter-observer agreement between radiologists was not assessed due to the retrospective design. Ultrasound findings were likewise recorded descriptively in routine clinical documentation without the use of a standardized research scoring system. In addition, no multivariable adjustment was performed because of the limited number of high-grade cases and outcome events, to avoid model overfitting.

Finally, the study was conducted in two surgical centers, and imaging workflows as well as emergency triage practices may vary across institutions and healthcare systems, potentially limiting the generalizability of the findings. Prospective multicenter studies integrating clinical, laboratory, and imaging parameters — including contrast-enhanced CT where appropriate — are warranted to further clarify the independent prognostic value of early morphologic assessment in acute pancreatitis.

## Conclusions

In this real-world cohort, ultrasound-first and CT-first imaging pathways in acute pancreatitis primarily reflected organizational and triage-related factors rather than intrinsic differences in imaging performance. Patients undergoing CT-first imaging demonstrated numerically higher rates of adverse outcomes, consistent with differences in early clinical context.

In patients undergoing non-contrast CT at admission, morphologic severity demonstrated consistent numerical gradients across clinical outcomes; however, statistical significance was not reached in dichotomized analyses. Early non-contrast CT morphology should therefore be interpreted as contextual inflammatory assessment rather than a standalone prognostic tool.

## Data Availability

The data supporting the findings of this study are available from the corresponding author upon reasonable request.
